# Spectral Entropy Based Neuronal Network Synchronization Analysis Based on Microelectrode Array Measurements

**DOI:** 10.3389/fncom.2016.00112

**Published:** 2016-10-18

**Authors:** Fikret E. Kapucu, Inkeri Välkki, Jarno E. Mikkonen, Chiara Leone, Kerstin Lenk, Jarno M. A. Tanskanen, Jari A. K. Hyttinen

**Affiliations:** ^1^Department of Pervasive Computing, Tampere University of Technology Tampere, Finland; ^2^Computational Biophysics and Imaging Group, Department of Electronics and Communication Engineering, BioMediTech, Tampere University of Technology Tampere, Finland; ^3^Department of Psychology, Center for Interdisciplinary Brain Research, University of Jyväskylä Jyväskylä, Finland; ^4^Department of Management and Production Engineering, Politecnico di Torino Torino, Italy

**Keywords:** synchronization, spectral entropy, correlation, mouse cortical cells, rat cortical cells, developing neuronal networks, MEA, microelectrode array

## Abstract

Synchrony and asynchrony are essential aspects of the functioning of interconnected neuronal cells and networks. New information on neuronal synchronization can be expected to aid in understanding these systems. Synchronization provides insight in the functional connectivity and the spatial distribution of the information processing in the networks. Synchronization is generally studied with time domain analysis of neuronal events, or using direct frequency spectrum analysis, e.g., in specific frequency bands. However, these methods have their pitfalls. Thus, we have previously proposed a method to analyze temporal changes in the complexity of the frequency of signals originating from different network regions. The method is based on the correlation of time varying spectral entropies (SEs). SE assesses the regularity, or complexity, of a time series by quantifying the uniformity of the frequency spectrum distribution. It has been previously employed, e.g., in electroencephalogram analysis. Here, we revisit our correlated spectral entropy method (CorSE), providing evidence of its justification, usability, and benefits. Here, CorSE is assessed with simulations and *in vitro* microelectrode array (MEA) data. CorSE is first demonstrated with a specifically tailored toy simulation to illustrate how it can identify synchronized populations. To provide a form of validation, the method was tested with simulated data from integrate-and-fire model based computational neuronal networks. To demonstrate the analysis of real data, CorSE was applied on *in vitro* MEA data measured from rat cortical cell cultures, and the results were compared with three known event based synchronization measures. Finally, we show the usability by tracking the development of networks in dissociated mouse cortical cell cultures. The results show that temporal correlations in frequency spectrum distributions reflect the network relations of neuronal populations. In the simulated data, CorSE unraveled the synchronizations. With the real *in vitro* MEA data, CorSE produced biologically plausible results. Since CorSE analyses continuous data, it is not affected by possibly poor spike or other event detection quality. We conclude that CorSE can reveal neuronal network synchronization based on *in vitro* MEA field potential measurements. CorSE is expected to be equally applicable also in the analysis of corresponding *in vivo* and *ex vivo* data analysis.

## Introduction

Temporally correlated activity between neurons or neuronal networks *in vivo* and *in vitro* has been vastly studied in terms of event based synchrony, or synchrony between oscillations or rhythmic activities in different frequency bands. Salinas and Sejnowski (2001) argued that the presence of correlations between the activities of pairs of neurons, or synchrony *per se*, is not important in general, since they may arise from common inputs or synaptic interactions, or from overlapping perceptive fields, respectively; however, changes in the correlation structure of a neuronal network reflect changes in its functional connectivity. Previous studies have shown that the pattern of synchronization determines the pattern of neuronal interactions, and that the efficiency of transferred information is also modulated by synchrony (Buehlmann and Deco, 2010; Battaglia et al., 2012). Thus, assessing the relations of synchrony is essential not only for fully developed neuronal networks, such as in the brain, but also for the assessment of development and plasticity of cultured neuronal networks.

In the past years, several studies concentrated on quantifying and analyzing the network relations of cultured neuronal cells (Garofalo et al., 2009; Mack et al., 2014). Most of the studies utilized binary analysis based on events, particularly the occurrences of spikes and bursts. For example, in several studies, transfer entropy (TE), joint entropy, mutual information (MI), coincidence index, and event synchrony (ES) were employed on detected spikes to evaluate network relations (Quiroga et al., 2002; Garofalo et al., 2009; Ito et al., 2011); however, as stated by Buzsáki et al. (2012), network relations affect local field potentials (LFPs) as well.

For brain studies, synchronization, causality, phase and frequency coupling, or tracking previously defined rhythms, can reveal network interactions (Ginter et al., 2005; Buehlmann and Deco, 2010). A review of a few connectivity measures to assess neuronal activity has been presented by Bastos and Schoffelen (2016). The amount of propagating activity observed in different frequency bands (rhythmic activities) or associated with well-defined electroencephalogram (EEG) rhythms (delta, theta, alpha, beta, and gamma) is important in interpreting the results of such a study (Ginter et al., 2005). On the other hand, generally in cultured neuronal networks, the different frequency bands are not as distinguishable as in the brain studies, or the absence of well-defined rhythms makes the analysis more challenging. Even though LFPs (or raw recordings that may include both spikes and LFPs) potentially carry information on network relations, they are not commonly used for the network analysis based on microelectrode array (MEA) measurement data from cultured cells. MEAs are usually used to measure extracellular field potentials from electrically active tissues and cell cultures at network and cell levels (Thomas et al., 1972; Gross et al., 1977; Pine, 1980; Egert et al., 1998). MEA electrodes record field potentials, e.g., from the neurons in their vicinity, which can carry contributions from both extracellular action potentials (EAPs) from individual neuronal cells and lower frequency contributions originating from neuronal population activity. Neurons may temporarily arrange themselves into synchronous functional ensembles to perform a given task. These ensembles may be volatile and only exist for short periods of time before new ensembles with partially different subsets of neurons are formed. Connected neuronal ensembles are thought to operate at certain frequencies (for general references, see Buzsáki and Chrobak, 1995; Penttonen and Buzsáki, 2003; Buzsáki and Draguhn, 2004). Consequently, frequency domain analysis has potential to obtain novel information also from cell cultures (Jarvis and Mitra, 2001; Brown et al., 2004). Frequency spectrum analysis may also be a good alternative in cases with unreliable spike detection either due to low amplitude spikes in noise or conflicting results from different spike detection algorithms.

Drawing from above, we have hypothesized that also temporal correlations of the frequency spectrum distributions could reflect the network relations of neuronal populations (Kapucu et al., 2016a). Intuitively, this is motivated by the possibility that measurements from functionally connected neuronal populations may be quite different if only time domain properties were considered. For analyzing the functional connectivity of a network, techniques quantifying the spectral properties of neuronal ensemble activity provide promising alternatives to the methods assessing the couplings or correlations between specific rhythms or frequencies. Spectral entropy (SE) quantifies the regularity, or complexity, of a signal based on its frequency dynamics. SE is a frequency based realization of Shannon's entropy algorithm (Shannon, 1948), which was previously used for analyzing certain neuronal events, such as burst suppression, and for the EEG based assessment of the depth of anesthesia (Viertiö-Oja et al., 2004). In our previous work (Kapucu et al., 2015), we utilized SE and sample entropy to quantify *in vivo* and *in vitro* neuronal bursts according to their complexities, and demonstrated similarities in the complexity values of bursts from neighboring channels. Also in Kapucu et al. (2016a), we tested the feasibility of SE for the assessment of synchronization. However, the method was never validated with simulations and its true applicability was not demonstrated with larger real measurement datasets. An earlier study of the relations between the synchronization and the activity level, as well as the relations between synchronization and connectivity levels (Chawla et al., 1999), was also a motivation for the evaluation of CorSE with different levels of connected networks.

In this paper, we expand the original idea proposed by Kapucu et al. (2016a) and investigate the benefits of SE time course correlation analysis as a tool for analyzing synchronization by analyzing a larger set of data. Here, we also name the proposed method CorSE. Firstly, we illustrate CorSE with a toy simulation of neuronal ensembles (Montgomery, 2014). Next, we validate our method with simulated MEA data produced with computational integrate-and-fire model based neuronal networks with known connectivity levels. The results of CorSE are compared to and assessed together with the results from three existing event based synchronization assessment algorithms, ES (Quiroga et al., 2002), MI (Gray, 1990), and TE (Schreiber, 2000). Finally, we demonstrate the applicability of CorSE with MEA data measured from cultured rat cortical neurons with different activity levels, and with MEA data measured from a developing network of mouse cortical neurons.

## Materials and methods

### Biological data

*In vitro* MEA experiments were performed with dissociated rat and mouse cortical cell cultures. The data from dissociated rat cortical cells was originally collected for a previous study (see the details given by Weihberger et al., 2013); animal treatment was according to the Freiburg University (Freiburg, Germany) and German guidelines on the use of animals in research. Briefly, rat cortical cells were obtained from prefrontal cortical tissue of newborn Wistar rats and plated on MEA plates, which consist of 60 titanium nitride electrodes of 30 μm diameter and 200 μm interelectrode spacing on an 8 × 8 rectangular grid with corner electrodes missing (model: 60MEA200/30iR, Multi-Channel Systems MCS GmbH, Reutlingen, Germany). Seeded cell density was approximately 1250 cells/mm^2^. After 4 weeks of culturing, the cultures were considered mature (Wagenaar et al., 2006) and recordings were conducted inside a dry incubator.

To assess *in vitro* network development over time, commercially available mouse cortical cells (A15586, Gibco, Thermo Fisher) were plated on MEAs similar to those described above. Briefly, the MEAs were coated with poly-L-lysine and laminin, and the cells were sowed as droplets on to MEA plates for culturing (Wagenaar et al., 2006). Electrophysiological data were recorded three times a week starting from 4 days *in vitro* (DIV) until the 29th DIV. Every recording lasted for 5 min.

The recordings from rat cortical cell cultures were analyzed using the different synchronization assessment methods considered in this paper. All *in vitro* data was first filtered with a 50 Hz notch filter and 7 Hz high pass filter to alleviate the powerline noise and low frequency fluctuations, respectively. For the analysis methods that are based on spike time stamps, spikes were detected using two thresholding methods to evaluate the effects of different spike detection methods on the results of the synchronization assessment algorithms: spike detection thresholds were set to five times the standard deviation of the signal, or at five times the estimated standard deviation of the background noise of the band pass filtered signal as proposed by Quiroga et al. (2004). Here, the different thresholding methods are denoted by STD and eSTD, respectively. CorSE was employed to demonstrate the tracking of network development in the mouse cortical cell cultures. Since CorSE operates on the measured signals themselves (either raw or filtered), spike detection was not performed.

Two cultures were selected for the analysis according to the number of active locations where spiking activity was observed according to a house made rule of 50 spikes per 300 s (Kapucu et al., 2012, 2016b) so that the chosen MEAs had different numbers of active locations. The analyzed cultures are denoted by MEA1 and MEA2. The MEA1 had 32 or 37 active sites as calculated with STD and eSTD, respectively, and MEA2 had 15 or 12 active sites as calculated with STD and eSTD, respectively.

### Simulated data

#### Toy simulations of correlated time-variant SEs

**Figure 1:**
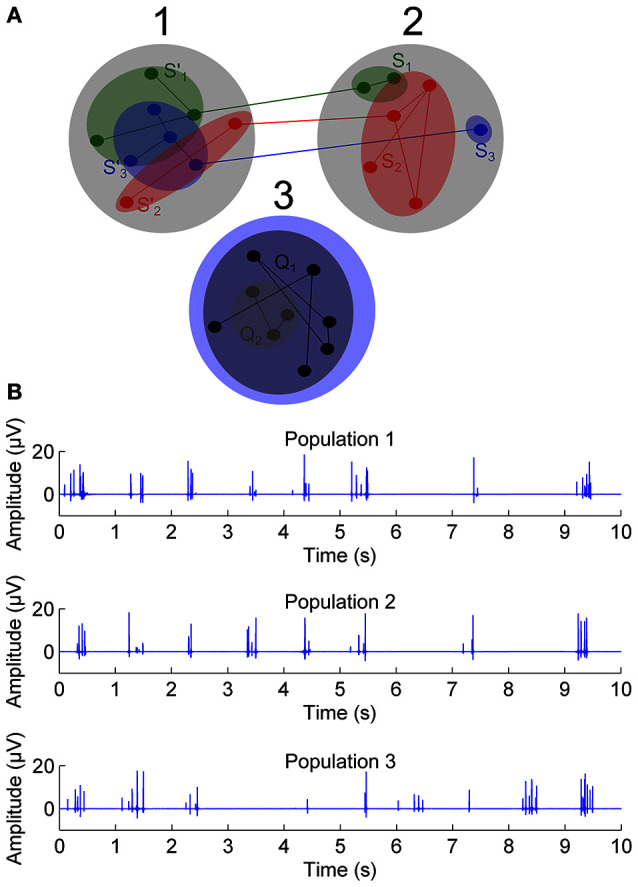
**The toy simulation of three populations with time correlated spectral complexities. (A)** Three populations; populations 1 and 2 have mutually correlated spectral complexities in time and population 3 not. Green, red, and blue color filled ellipses are illustrated neuronal ensembles. **(B)** Sample 10 s simulations exhibiting EAPs, from 1st, 2nd, and 3rd populations.

A toy simulation of a MEA signal was generated to illustrate and explain the proposed SE based synchronization assessment method CorSE. Here, MEA measured neuronal unit activities, i.e., EAPs, and the average activity of neuronal ensembles, i.e., LFPs, were simulated as cardinal *Sine*, i.e., *Sinc* waves, and oscillation of *Sines*, respectively (see Montgomery, 2014 for the *Sine* wave representation of LFPs), to generate a simulated population signal as seen via an electrode. In such a model, neuronal synchronization between two populations was defined as simultaneous activity of neuronal ensembles in a simple way: when a number of neuronal ensembles (illustrated with green, red, and blue color filled ellipses in Figure 1A) activated in a population, the same number of neuronal ensembles was also activated in the other population, i.e., the model assumes that all the active neuronal ensembles are connected to other ensembles in the other population. Three populations were simulated with two of them (populations 1 and 2) fully synchronous with each other, and the third population (population 3) independent of the other populations (Figure 1A).

Simulated signals were generated in 1 s sections which were simply concatenated to form a simulation of a 3 min measurement. For each 1 s section, a random number of constituent *Sine* signals, and a random number of *Sinc* signals, were generated and summed together. The sampling rate for the generated signals was 1 kHz. The number of constituent *Sine* signals was evenly distributed between 5 and 10, and the number of constituent *Sinc* signals evenly distributed between 0 and 10. The oscillation amplitudes, frequencies, and phases were randomly selected and evenly distributed. To generate the two connected populations, whenever a *Sine* or *Sinc* appeared in one of them, a *Sine* or *Sinc*, respectively, appeared also in the other population, both with independently random amplitudes, frequencies, and phases. The signals for the unconnected population were generated independently of the other two populations.

The simulations were implemented in Matlab (MathWorks, Inc., Natick, MA, USA). A large data set of 1000 triplets of populations 1, 2, and 3 was generated, each simulated signal corresponding to a 3 min recording. To investigate the functioning of the methods with either LFPs or EAPs or both, signals with five different EAP-LFPs power ratios *P*_*EAPs*/*LFPs*_ = 0, 10, 20, 50, and 100% were generated (*P*_*EAPs*_ and *P*_*LFPs*_ denote the total estimated powers of the summed constituent *Sinc* and *Sine* signals, respectively).

Statistical validation was approached by calculating all pairwise synchronizations between all 1000 simulated recordings in all three populations, and by calculating statistics for the signals from the two connected populations to express detected synchronization vs. the synchronization between signals from the unconnected populations.

#### Simulations with integrate-and-fire model based neuronal networks

The computational neuronal network simulation was based on the model introduced by Tsodyks et al. (2000). The networks consisted of integrate-and-fire neurons with short-term plastic synapses, and exhibited clear population bursts. The parameters employed for neurons and synapses were the same as in Tsodyks et al. (2000). To introduce spontaneous activity in the network, the neurons were driven with white noise current. Three-minute MEA measurements were simulated.

The simulated networks consisted of two populations. Each population consisted of 50 neurons, of which 40 were excitatory and 10 inhibitory. Each population was internally fully connected, but without autapses. Simulations were conducted at five inter-population connectivity levels, 0, 10, 20, 50, and 100%, between the two populations. Hundred percent connected populations correspond to one population twice the size of the original populations. Here, the percentages give the probabilities for one neuron to be connected to another neuron in the other population. Hundred pairs of populations were simulated for each inter-population connectivity level. The weights of all connections were tuned so that the mean spike rate in a population would not vary highly between the simulations with different levels of connectivity, resulting in the mean spike rate of 39–45 spikes/second in a simulation.

**Figure 2:**
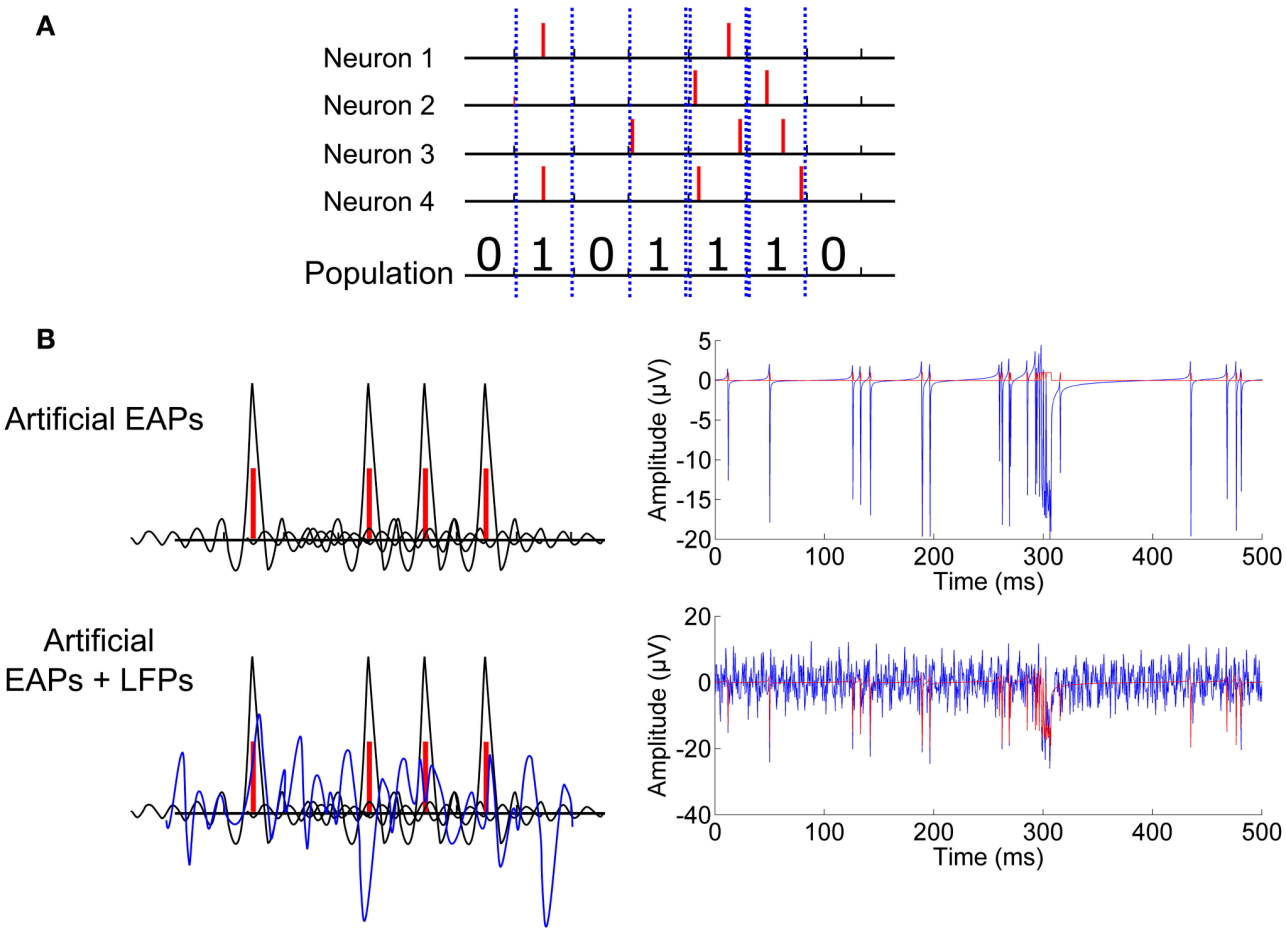
**Computational neuronal network simulation. (A)** Any number of spikes in a time bin is counted as one population spike in that time bin. **(B)** A *Sinc* function with random parameters (black) is placed at each population spike (red) forming the EAPs. Thereafter, artificial LFPs are simulated by additive *Sine* functions with random parameters. Artificially created EAPs with and without LFPs for population signaling are plotted with blue. Illustrative signal construction and the resulting exemplary signals can be seen on the left and right-hand panels respectively.

The NEST simulator (Gewaltig and Diesmann, 2007) was used to provide spike time stamps of the EAPs of the individual neurons. From the time stamps, artificial MEA recordings were constructed to simulate raw MEA recordings: the time resolution of the simulation was 1 ms, and a single spike was recorded for a one-millisecond time bin if any number of individual spikes appeared during the bin (Figure 2A). Thereafter, a *Sinc* function with random parameters, similarly as in the toy simulation, was formed for each spike and located in time with the maximum at the spike time point. The generated *Sinc* signals, peaking in general at different points in time, were summed to generated an EAP signal. Basically, a *Sinc* kernel can be used to reconstruct a sampled signal (Blanche and Swindale, 2006) or a population activity (Nawrot et al., 1999), but here we intended not to reconstruct the original recording from its samples, but instead to obtain a continuous function based on the simulated spike time stamps. We call these simulated signals artificial raw recordings (c.f., Figure 2B).

Artificial LFPs were added to the EAP simulations as described in Section CorSE, SE Based Synchronization Analysis (c.f., Figure 2B). Simulations were conducted at *P*_*EAPs*/*LFPs*_ ≈ 20% and *P*_*EAPs*/*LFPs*_ ≈ 50%, to roughly correspond to two difference scenarios of background noise and activity levels vs. action potential amplitudes. Thereafter, to make the simulations more realistic, white Gaussian noise (WGN) was added to the generated artificial raw signals for all cases simulated. WGN was added to obtain signal to noise ratios (SNRs) of 50% and 20%, as calculated by *SNR* = 100 · *P*_*EAPs*_/*P*_*WGN*_, where *P*_*EAP*_ is the estimated power of a generated artificial recording, and *P*_*WGN*_ is the estimated power of the added WGN.

### CorSE, SE based synchronization analysis

#### Shannon entropy

Entropy in the context of information theory was introduced by Shannon as a measure of uncertainty (Shannon, 1948). Shannon entropy is defined as

(1) $$H=-\sum_{i}p_{i}\log p_{i},$$

where *p*_*i*_ is the probability that an amplitude value occurs in the *i*th amplitude bin, and is given by the probability density function of the time series.

#### SE

We calculated SE as Shannon's entropy on power spectrum as described by Viertiö-Oja et al. (2004): SE was calculated by first obtaining the frequency spectrum of the time series ***x***(*n*), sampled at discrete time points *n*, by fast Fourier transform ***X***(*f*) at frequency points *f* (2) with bold denoting a vector.

(2) $$X\left(f\right)=\sum_{n}x(n)e^{-i2\pi fn}$$

The power spectrum is given by

(3) $$P\left(f\right)=X\left(f\right)X^{*}\left(f\right),$$

where X^*^(*f*) is the complex conjugate of ***X***(*f*). Here, power spectrum was estimated by Welch periodogram with a Hann window of a preset length of 0.5 s and 50% window overlap to provide a smoother transition between windows and to increase temporal resolution.

Power spectrum was normalized with a constant *C* for the Nyquist frequency range at *K* frequency points [*f*_1_, …, *f*_*k*_, …, *f*_*K*_] so that the sum of the normalized power spectrum ***P***_***norm***_(*f*_*k*_) equaled unity (4).

(4) $$\underset{f_{k=}f_{1}}{\sum^{f_{K}}}P_{norm}\left(f_{k}\right)=C\underset{f_{k=}f_{1}}{\sum^{f_{K}}}P\left(f_{k}\right)=1$$

SE *S* was calculated from the normalized power spectrum as

(5) $$S=\;\underset{f_{k=}f_{1}}{\sum^{f_{K}}}P_{norm}\left(f_{k}\right)\;\log\left(\frac{1}{P_{norm}\left(f_{k}\right)}\right),$$

and *S* was normalized to reside between 1 and 0 by

(6) $$S_{norm}=\;\frac{S}{\log\left(K\right)}$$

In the sequel, SE is always considered to be the normalized SE *S*_*norm*_.

#### Correlation of SEs

Here, we quantify the synchronization of signals by the correlation of the temporal changes of their spectral contents. This is obtained by calculating SEs in time windows, and the degree of common temporal changes for different sites in the neuronal network is assessed by calculating cross correlations: the cross covariance *C*_*S*_*x*___*S*_*y*__(*t, t* + τ) of the SEs ***S***_***x***_ and ***S***_***y***_ of the signals ***x*** and ***y***, respectively, describes how well the SE of the signal ***y*** at time *t* + τ is correlated with SE of the signal *x* at time *t*. Here, the sample cross correlations were estimated using the *crosscorr* function of Matlab (Chatfield, 2003). With *O* the number of time windows in which SEs were calculated, and *i* the index of a 0.5 s long time window, {(***S***_***x, i***_, ***S***_***y, i***_); *i* = 1, 2, …, *O*}, cross covariance at the lag *l* = 0 is given by

(7) $$C_{S_{x}S_{y}}=\;\frac{1}{O}\underset{i=1}{\sum^{O}}\left(\left(S_{x,i}-\bar{S_{x}}\right)\left(S_{y,i}-\bar{S_{y}}\right)\right),$$

where $\bar{S_{x}}$ and $\bar{S_{y}}$ are the sample means of the corresponding SEs. The cross correlation *r*_*S*_*x*___*S*_*y*__ at lag *l* = 0 is then estimated as

(8) $$r_{S_{x}S_{y}}=\frac{C_{S_{x}S_{y}}}{\sigma_{S_{x}}\sigma_{S_{y}}},$$

where σ_*S*_*x*__ and σ_*S*_*y*__ are the standard deviations of the corresponding SEs. SE cross correlation (8) values at lag zero of the were used to assess the level of synchronization between pairs of channels, i.e., CorSE between signals ***x*** and ***y*** is given by *CorSE*_*xy*_ = *r*_*S*_*x*_*S*_*y*__.

### Known event based methods used for comparison

For comparisons with CorSE, we implemented three commonly used event based synchronization assessment algorithms: ES (Quiroga et al., 2002), MI (Gray, 1990), and TE (Schreiber, 2000). Comparisons were made based on the results of all the algorithms considered for the computational neuronal network simulations and for the rat cortical cell recordings. EAPs detectable in these signals were taken as the events for the three event based algorithms considered. The spikes, i.e., EAPs, in both the artificial recordings and in real MEA recordings were detected with two different threshold based spike detection methods, STD and eSTD, as described in Section Biological Data, resulting in sets of binary strings as the inputs to algorithms. With the artificial recordings, the effects of added LFPs and WGN on the spike detection, and the consequences on the event based synchronization assessment algorithms, were also evaluated.

ES, introduced by Quiroga et al. (2002), measures synchronization based on quasi-simultaneous appearance of events. As an initial step, the ES algorithm finds from the time series ***x*** and ***y*** the maximum time period **τ**_***i, j***_ between two consecutive spikes so that the two spikes occurring at times ${\text{t}}_{\text{i}}^{\text{x}}$ and ${\text{t}}_{\text{j}}^{\text{y}}$ in signals ***x*** and ***y***, respectively, where i and j are spike indexes, can be considered simultaneous, by calculating the local spike appearances:

(9) $$\tau_{i,j}=\frac{\text{min}\left\{t_{i+1}^{x}-t_{i}^{x},t_{i}^{x}-t_{i-1}^{x},\;t_{j+1}^{y}-t_{j}^{y},t_{j}^{y}-t_{j-1}^{y}\right\}}{2}.$$

Then, cross covariance ***C***_***x|y***_ is defined as the number of times a spike appears in ***x*** within **τ**_***i, j***_ after a spike has appeared in ***y***, as:

(10) $$C_{x|y}=\;\underset{i=1}{\sum^{M_{x}}}\underset{j=1}{\sum^{M_{y}}}J_{i,j},$$

where *M*_*x*_ and *M*_*y*_ are the total numbers of events for ***x*** and ***y***, respectively, and

(11) $$J_{i,j}=\{\begin{array}{c} 1,0<t_{i}^{x}-t_{j}^{y}<\tau_{i,j}\\ \frac{1}{2},\;t_{i}^{x}=t_{j}^{y}\\ 0,\text{otherwise}. \end{array}$$

Finally, synchronization is given by

(12) $$Q=\frac{C_{x|y}+C_{y|x}}{\sqrt{M_{x}M_{y}}}.$$

The algorithm in its original form did not have a requirement for the minimum number of events to consider; thus, two time series with even one simultaneous detectable event could be considered as fully synchronized. To circumvent this for MEA recordings, we employed the in-house criterion of minimum 50 spikes in 300 s, as in Kapucu et al. (2012) and Kapucu et al. (2016b), for the data to be analyzed. This eliminates unjustified synchronizations, which could be caused by coincidentally appearing rare events. We named the modified algorithm the corrected ES (cES) method.

The next method we used for comparisons was the well-known mutual information (MI) (Gray, 1990), which has been widely employed in many studies to quantify dependencies between time series or specifically for quantifying synchronization (Garofalo et al., 2009; Salazar et al., 2012). MI (13) is calculated by considering both single and joint probabilities of events in two time series *x* and *y*.

(13) $$MI_{xy}=\;\sum_{e_{i}^{y}\in y}\sum_{e_{i}^{x}\in x}p(e_{i}^{x},e_{i}^{y})\log\left(\frac{p(e_{i}^{x},e_{i}^{y})}{p(e_{i}^{x})p(e_{i}^{y})}\right),$$

where $e_{i}^{x}$ and $e_{i}^{y}$ are *i*th single events occurring in the signals *x* and *y*, respectively, $p\left(e_{i}^{x},e_{i}^{y}\right)$ is the joint probability density function, and $p\left(e_{i}^{x}\right)$ and $p\left(e_{i}^{y}\right)$ are the single probabilities. Mutual information is a symmetric measure, i.e., *MI*_*xy*_ = *MI*_*yx*_.

The last algorithm is transfer entropy (TE), which extends the concept of MI to conditional properties by considering the history of the influenced information (Schreiber, 2000). In other words, *TE*_*y*→*x*_ measures the increase in predictability of knowing the future and the past of ***x***, once ***y*** is known. TE can be calculated as

(14) $$TE_{y\to x}=\;\sum_{e_{i}^{x},e_{i}^{y}}p\left(e_{i+1}^{x},e_{i}^{x},e_{i}^{y}\right)\log\left(\frac{p\left(e_{i+1}^{x}|e_{i}^{x},e_{i}^{y}\right)}{p\left(e_{i+1}^{x}|e_{i}^{x}\right)}\right),$$

where in addition to the parameters defined above, $p\left(e_{i+1}^{x}|e_{i}^{x}\right)$ denotes the conditional probability of observing the state $e_{i+1}^{x}$ after $e_{i}^{x}$.

TE (15) was calculated in one-millisecond signal bins for delays up to three bins, and the maximum value of TE was considered as given in Ito et al. (2011).

(15) $$TE_{y\to x}=\;\sum_{e_{i}^{x},e_{i}^{y}}p\left(e_{i+1}^{x},e_{i}^{x},e_{i+1-d}^{y}\right)\log\left(\frac{p\left(e_{i+1}^{x}|e_{i}^{x},e_{i+1-d}^{y}\right)}{p\left(e_{i+1}^{x}|e_{i}^{x}\right)}\right),$$

where *d* is the time delay. Since TE is asymmetric and we did not consider the directionality, we considered the maximum TE value obtained between the channel pairs, i.e., *TE* = *max*(*TE*_*y*→*x*_, *TE*_*x*→*y*_).

### Assessment of the toy simulations

To present the results of the toy simulation, it is necessary to define the detection of correct synchronization, false positive synchronization, and false negative synchronization, and to calculate their rates. Here, synchronization is detected correctly if CorSE between populations 1 and 2 is larger than CorSE between populations 2 and 3, and larger than between populations 1 and 3, i.e., when *CorSE*_*XY*_ > *CorSE*_*XZ*_ and *CorSE*_*XY*_ > *CorSE*_*YZ*_. In this case, populations 1 and 3, and populations 2 and 3 are not deemed synchronized. False negative synchronization is detected when the true synchronization is missed, i.e., when either CorSE between populations 1 and 3, or between populations 2 and 3, is greater than or equal to CorSE between populations: *CorSE*_*XY*_ ≤ *CorSE*_*XZ*_ and/or *CorSE*_*XY*_ ≤ *CorSE*_*YZ*_. False positive synchronization is detected when either populations 1 and 3, or populations 2 and 3 are deemed synchronized. In all cases of false positive detection, the true synchronization is also missed. Vice versa, in all cases of false negative synchronization, a false positive synchronization is detected. Thus, due to the system of populations in Figure 1, and the synchronization criterion applied, the false positive and false negative rates are equal.

### Comparative assessment of the analysis methods for MEA recording analysis

For comparing the synchronization values obtained with the different methods considered, we first calculated the synchronization between each electrode pair, and thereafter created adjacency matrixes of the synchronization values. In **Figures 5, 6**, the matrixes are arranged for better visualization according to the ascending channel numbers on a MEA plate, e.g., channel 12 is located at (1,2) on an 8 × 8 MEA layout. In each matrix, the values were normalized respect to the maximum of the matrix to form a color scale. This was done to make the locations of the maximally synchronized electrode pairs more easily comparable between the different methods.

Since the signals were recorded from unguided neuronal cells that freely form networks on the MEA plates, there was no ground truth available on the actual connections or synchronization between the neuronal populations. However, we compared the results from the different algorithms based on a fixed number of most synchronized channel pairs according to each algorithm, i.e., the strongest synchronizations; to illustrate the differences in the results of difference algorithms, the 40 strongest synchronizations were found using each algorithm for MEA1, and the 20 strongest synchronizations for MEA2.

Also, we evaluated the similarity of the synchronization based functional connectivity results of the algorithms. Here, we considered the measurement channels as nodes and synchronized channel pairs as links. The results are presented as the numbers of links and nodes common between the results from the different methods, when considering only 10, 20, …, or 50 strongest synchronizations found with each algorithm, i.e., synchronization maps were drawn with each method, and similar findings between pairs of methods were reported by plotting the numbers of the found common nodes and the numbers of common links as functions of the number of the strongest synchronizations considered.

## Results

### Toy simulation analysis results

The Results from the analysis of the toy simulations with 1000 triplets indicate that CorSE was able to clearly distinguish the two populations with time correlated frequency distributions from the population pairs that did not have the correlated frequency distributions by design. In Table 1, results are shown for the model with LFPs, with EAPs, and for a model with both LFPs and EAPs with different *P*_*EAPs*/*LFPs*_ s (see Section SE). It is seen from Table 1 that over 96% of the cases were correctly identified as synchronized, and the results show consistent performance regardless of the EAP-LFP power ratio considered. In summary, the results in Table 1 demonstrate that the method can detect synchronization in the case simulated.

**Table 1:** **Synchronization detection rates based on 1000 simulation of the toy model signal analysis with different EAP-LFP power ratios**.

**Simulated EAP-LFP power ratio**	**Synchronization detection rate (%)**	**False positive rate; false negative rate (%)**
*P_EAPs/LFPs_* ≈ 100% (EAPs only)	99.8	0.2
*P_EAPs/LFPs_* ≈ 50%	97.9	2.1
*P_EAPs/LFPs_* ≈ 20%	97.1	2.9
*P_EAPs/LFPs_* ≈ 10%	96.7	3.3
*P_EAPs/LFPs_* ≈ 0% (LFPs only)	99.5	0.5

***X**, **Y**, and **Z**, denote the recorded signals from the populations 1, 2, and 3 respectively, with only **X** and **Y** with mutually correlated frequency distributions. For this analysis system, false positive rate is equal to false negative rate*.

### Integrate-and-fire model simulation analysis results

We assessed the simulated integrate-and-fire model based computational neuronal networks with CorSE and compared the results with the three different event based synchronization assessment algorithms described in Section Known Event Based Methods Used for Comparison. Firstly, in Table 2, we present the spike detection results employing two different spike detection methods, i.e., STD and eSTD, and with respect to the different EAP-LFPs power ratios considered, as well as different with levels of added WGN. Table 2 shows the approximate mean values in percentages for false negative (FN) detections, i.e., the missed spikes for all 1000 artificial recordings created from 100 simulations for each five connectivity levels. False positive detection, i.e., detected spurious spikes, were observed very rarely and could be considered negligible in all cases. Results are given for the both spike detection methods for *P*_*EAPs*/*LFPs*_ ≈ 20 and 50%, as well as for SNRs of 20 and 50%. The results in Table 2 indicate that both additive LFPs and WGN greatly affect spike detection, which is natural; the phenomenon occurs in all thresholding based spike detection systems, since with increasing LFP and/or WGN power, more and more spikes fall below overall noise level and cannot be detected by thresholding, and subsequent analysis is done based on only the detectable spikes. In practice in general, the FN detections correspond to the action potentials from neurons far away from the measurement electrode, so that the action potential amplitudes fall below the general noise level; such neurons are naturally more abundant than the neurons in the close vicinity of the electrode.

**Table 2:** **Spike detection performances using STD and eSTD for the artificially generated MEA signals**.

**Spike detection method**	**Added signal**	***P_EAPs/LFPs_* (for LFPs); SNR (for WGNs) (%)**	**False negative rate (%)**
STD	LFP	~50	64
		~20	97
	WGN	50	90
		20	99
eSTD	LFP	~50	45
		~20	96
	WGN	50	87
		20	99

**Figure 3:**
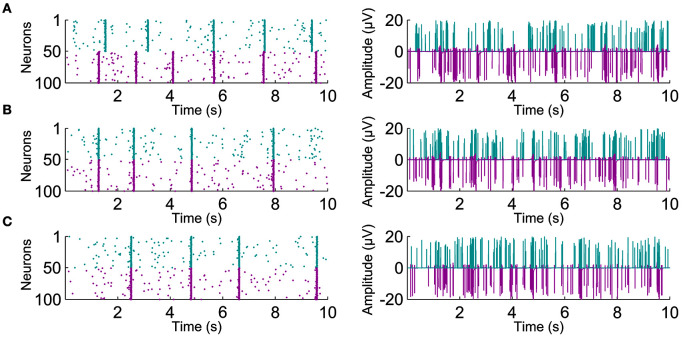
**Exemplary raster plots with the increasing connectivity**. Left-hand panels show individual spike activity of 100 neurons for 10 s. Spikes from the two different populations are shown with different colors. Right-hand panels show the artificial recordings calculated from the corresponding population spike activity. The artificial recordings are shown for **(A)** 0%, **(B)** 50%, and **(C)** 100% neuronal connectivity.

**Figure 4:**
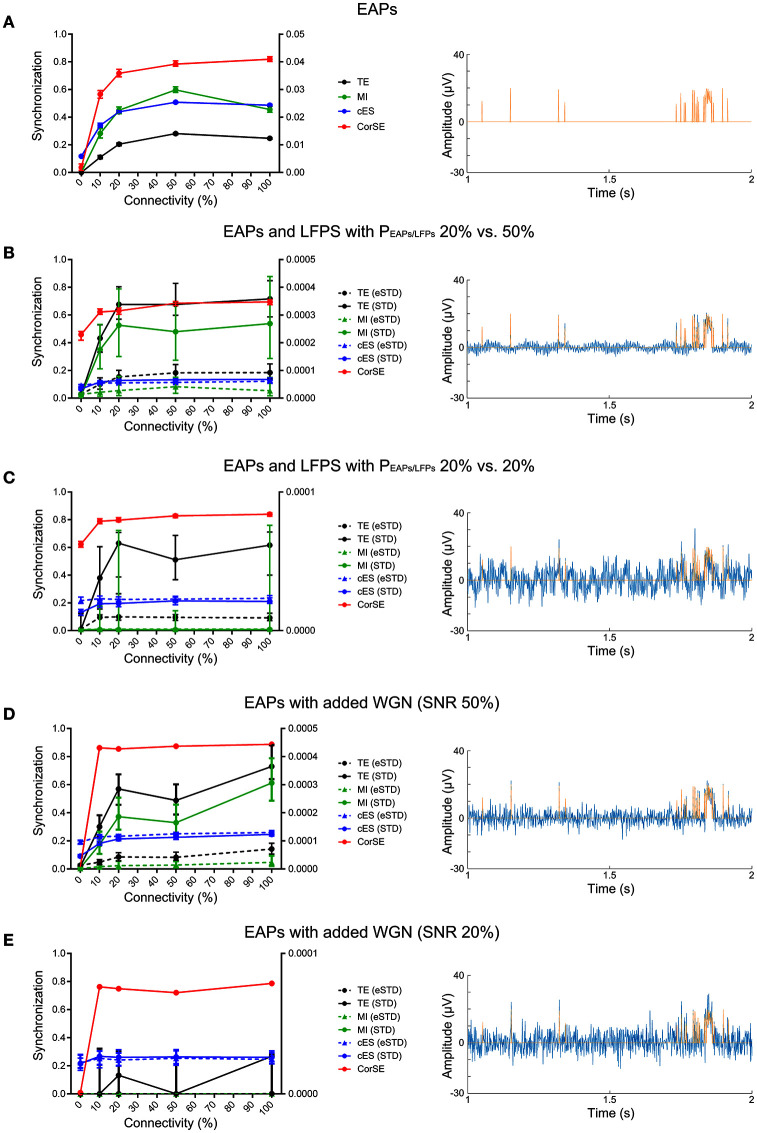
**The synchronization results by the different algorithms for the different connectivity levels**. Left-hand panels present the synchronicities calculated for the artificial recordings by CorSE (red) and cES (blue) methods (left vertical axis), and TE (black) and MI (green) methods (right vertical axis). For the event based methods, the line types indicate the spike detection method used: STD (solid), and eSTD (dashed). The right-hand panels show exemplary 2 s signal segments with EAPs (orange), and LFP or WGN (blue) signal components. The left-hand panels: **(A)** Synchronicities calculated from the artificial recordings with only EAPs. **(B)** The synchronicities calculated from the artificial recordings with both EAPs and LFPs with *P*_*EAPs*/*LFPs*_ ≈ 50 % for one population and *P*_*EAPs*/*LFPs*_ ≈ 20 % for the other. **(C)** Synchronicities calculated from the artificial recordings with both EAPs and LFPs with *P*_*EAPs*/*LFPs*_ ≈ 20 % for both populations. **(D)** Synchronicities calculated from the artificial recordings with EAPs and added WGN with *SNR* = 50 %. **(E)** The synchronicities calculated from the artificial recordings with EAPs and added WGN with *SNR* = 20 %.

Next, we evaluated the synchronization values obtained by the different algorithms for the different connectivity levels. Exemplary raster plots for individual neurons and their corresponding artificial population activity for different connectivity levels are presented on the left-hand and right-hand panels of Figure 3, respectively. By visual inspection, inter- and intra-population synchronization can be observed in the left-hand panels of Figure 3. In Figure 4, we present the calculated synchronicities from the artificial recordings in the left-hand panels, and the corresponding sample signals in the right-hand panels. Figure 4A presents the synchronicities calculated from the artificial recordings with only EAPs (see the right-hand panel for an exemplary 2 s signal segment). Synchronization values calculated by all the algorithms increase with the increasing connectivity, except for the connectivity levels 50 and 100% for all other methods than CorSE. CorSE was the only method showing monotonous increase in the synchronization when the connectivity increased for the considered connectivity values.

Figures 4B,C presents the synchronicities calculated from the artificial recordings with EAPs and added fully synchronized LFPs. Comparisons between the results with the EAP-LFP power rations *P*_*EAPs*/*LFPs*_ ≈ 20*% vs*. 50%, and *P*_*EAPs*/*LFPs*_ ≈ 20*% vs*. 20% can be seen in Figures 4B,C, respectively. With the added artificial LFPs (see Figures 4B,C right-hand panels for exemplary 2 s signal segments with *P*_*EAPs*/*LFPs*_ ≈ 50 *and* 20%, respectively), the synchronization levels detected with the event based algorithms were noticeably smaller than those detected with CorSE as predictable from the FN spike detection rates shown in Table 1. With LFPs or WGN (Figures 4B–E), it is observed that the increased connectivity did not always lead to increased observed synchronization. Results indicate that the spike detection performances have a strong influence on the synchronization results of the event based algorithms, as expected. In contrary, although synchronization values of the artificial recordings measured with CorSE changed with the superimposed LFPs, the correlation between the increased synchronization and the increased level of connectivity is somewhat preserved and the behavior between simulations remained similar.

Figures 4D,E shows the synchronicities calculated from the artificial recordings with EAPs and added WGN. The results from the different SNR values, 50 and 20%, are presented in Figures 4D,E, respectively, whereas right-hand panels again show corresponding exemplary 2 s signal segments. Adding WGN greatly decreased the synchronization detected by the event based algorithms (c.f., Figures 4D,E). On the other hand, to compare, cES presented better performance in distinguishing the relation between the increasing levels of connectivity and synchronization. Synchronization values calculated by CorSE changed much less due to WGN than those for the event based methods. Even though CorSE was still able to distinguish the unconnected (0% level connectivity) populations from the connected populations, it cannot distinguish the different levels of connectivity; especially with the lower SNR.

In conclusion, CorSE was applicable in determining the level of synchronization according to the level of population connectivity not only for the simulated recordings which solely consisted of EAPs, but also in presence of LFPs. CorSE was also usable for functional connectivity assessment to distinguish the connected and unconnected populations in low SNR conditions caused by high WGN power.

### MEA recordings

**Figure 5:**
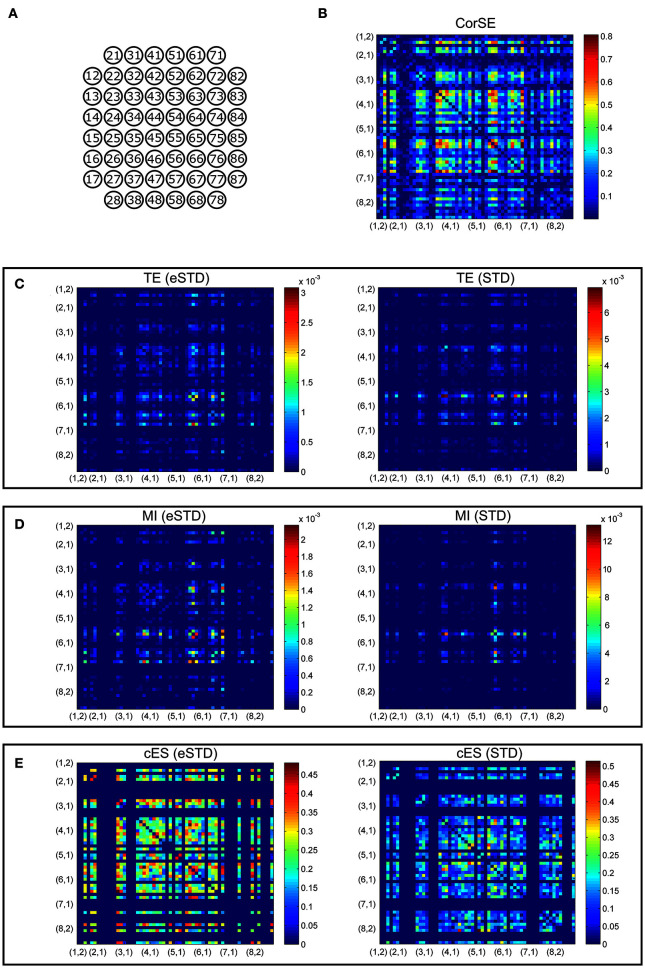
**Adjacency matrixes of the synchronization values given by the different algorithms for MEA1. (A)** MEA layout. The synchronization values calculated with **(B)** CorSE, **(C)** TE with eSTD (left) and with STD (right), **(D)** MI with eSTD (left) and with STD (right), and **(E)** cES with eSTD (left) and with STD (right). The color scales are normalized for each matrix separately regarding to the maximum value obtained.

**Figure 6:**
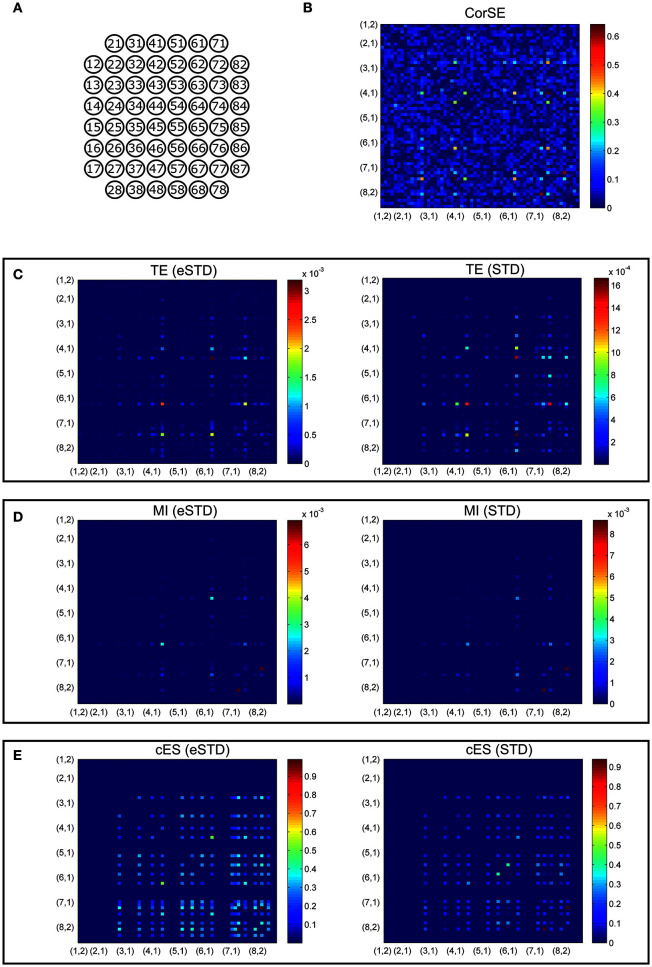
**Adjacency matrixes of the synchronization values given by the different algorithms for MEA2. (A)** MEA layout. The synchronization values calculated with **(B)** CorSE, **(C)** TE with eSTD (left) and with STD (right), **(D)** MI with eSTD (left) and with STD (right), and **(E)** cES with eSTD (left) and with STD (right). The color scales are normalized for each matrix separately regarding to the maximum value obtained.

**Figure 7:**
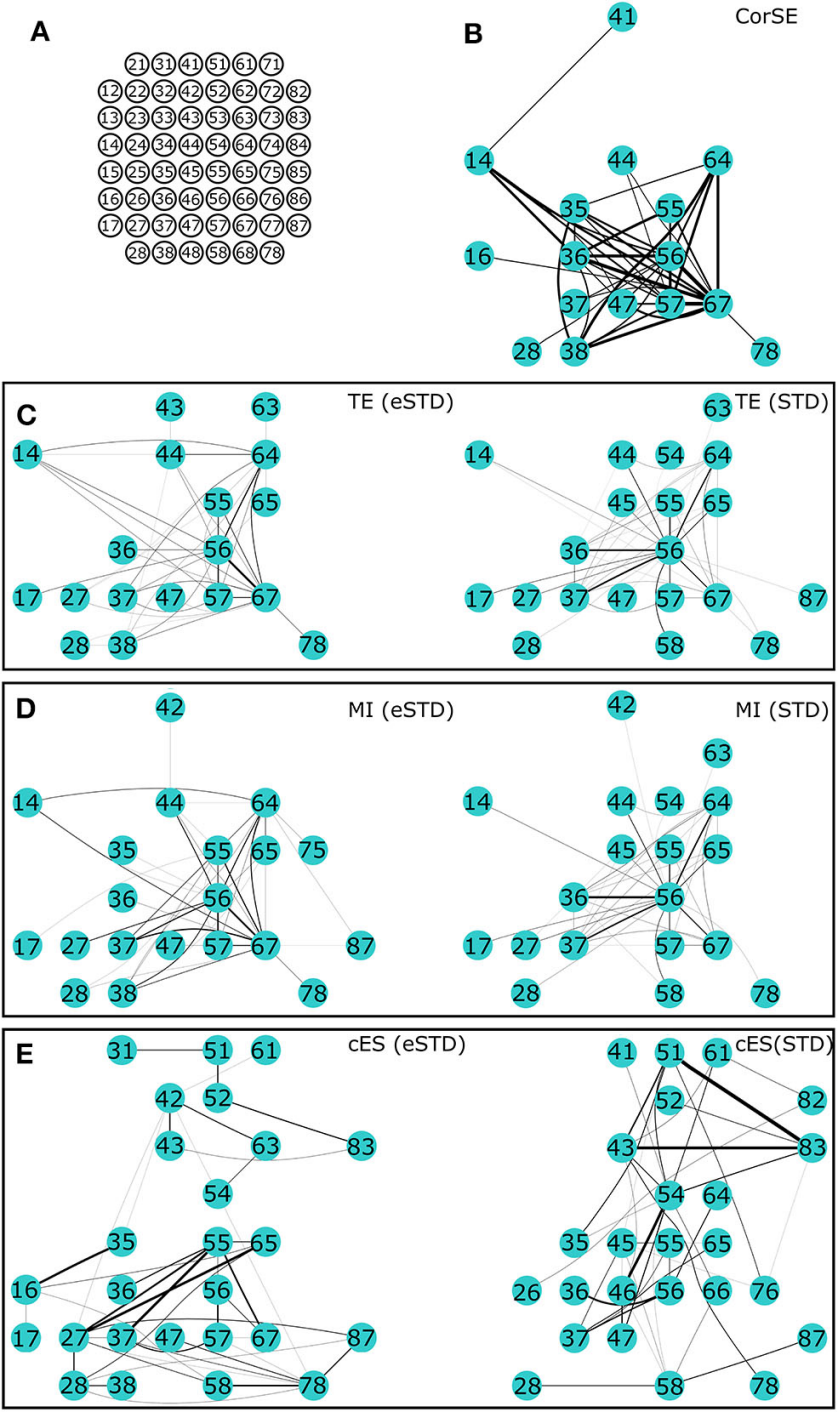
**The 40 most synchronized channel pairs (strongest synchronizations) calculated for MEA1. (A)** MEA layout. The strongest paired channels and their links found by **(B)** CorSE, **(C)** TE with eSTD (left) and with STD (right), **(D)** MI with eSTD (left) and with STD (right), and **(E)** cES with eSTD (left) and with STD (right).

**Figure 8:**
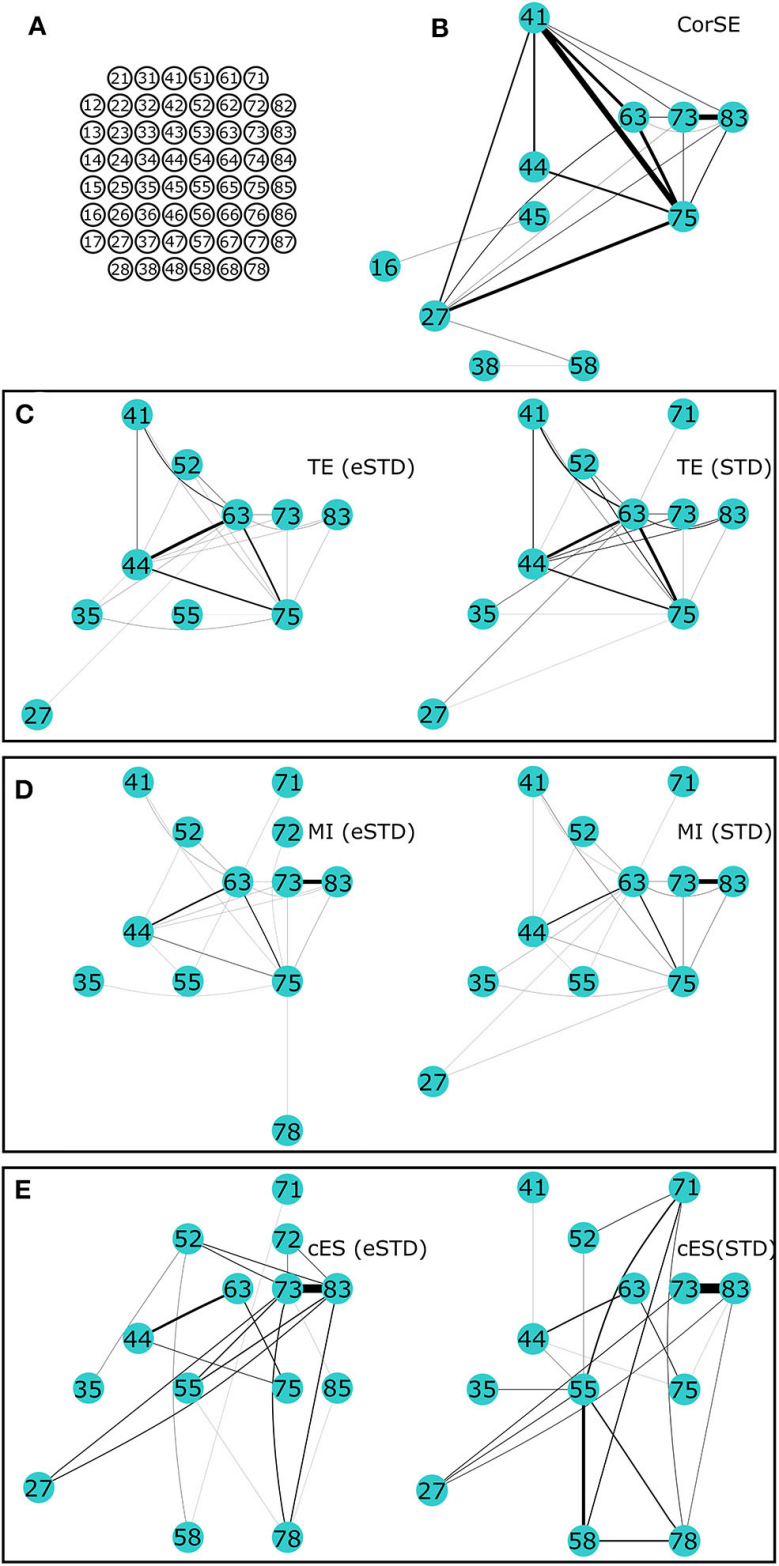
**The 20 most synchronized channel pairs (strongest synchronizations) calculated for MEA2. (A)** MEA layout. The strongest paired channels and their links found by **(B)** CorSE, **(C)** TE with eSTD (left) and with STD (right), **(D)** MI with eSTD (left) and with STD (right), and **(E)** cES with eSTD (left) and with STD (right).

**Figure 9:**
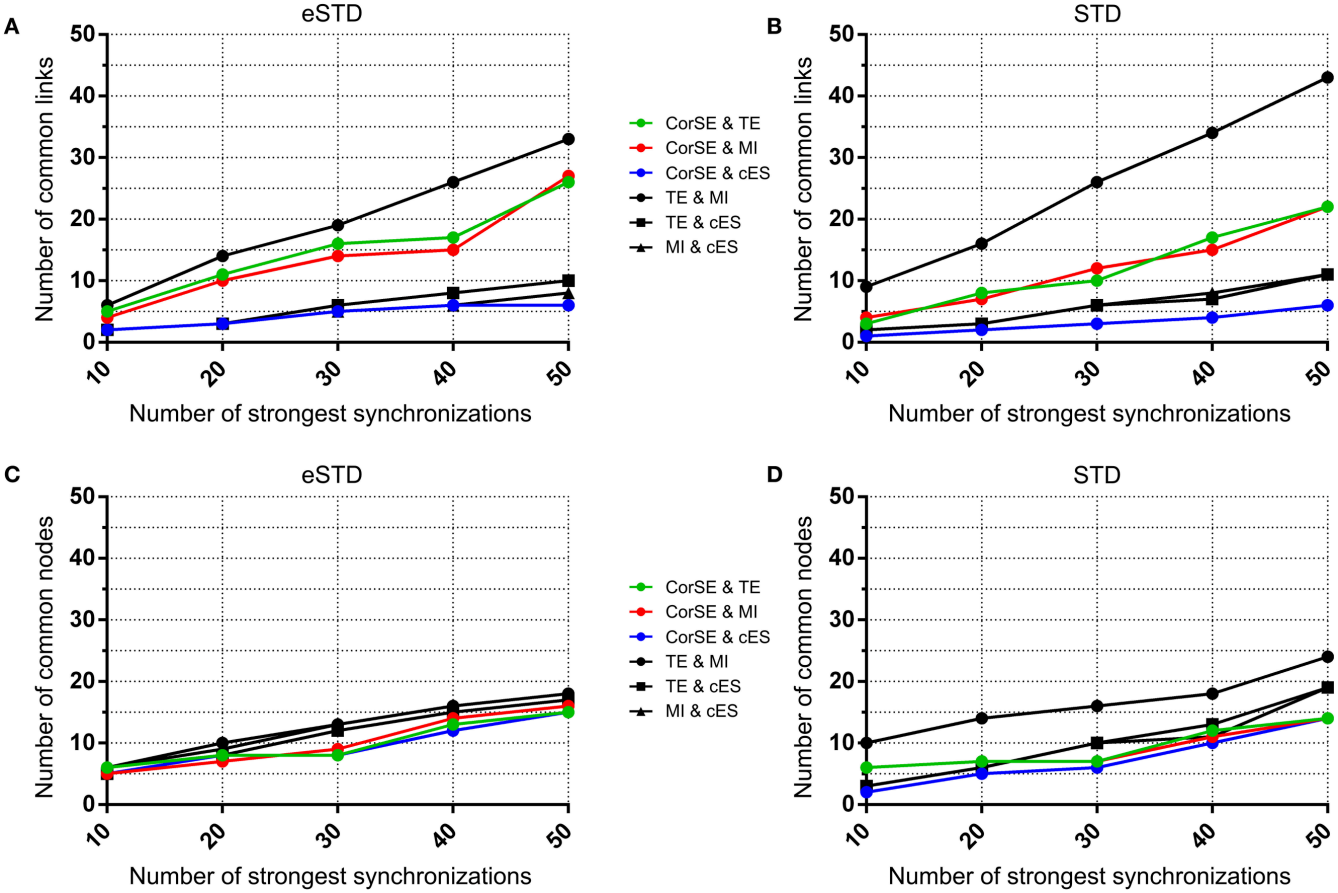
**The number of common nodes (channels) and the number of common links (pairwise synchronizations) as a function of the number of synchronizations for MEA1**. The number of common links found by the different algorithms using **(A)** eSTD and **(B)** STD based spike detection. The number of common nodes found by different algorithms using **(C)** eSTD and **(D)** STD based spike detection.

**Figure 10:**
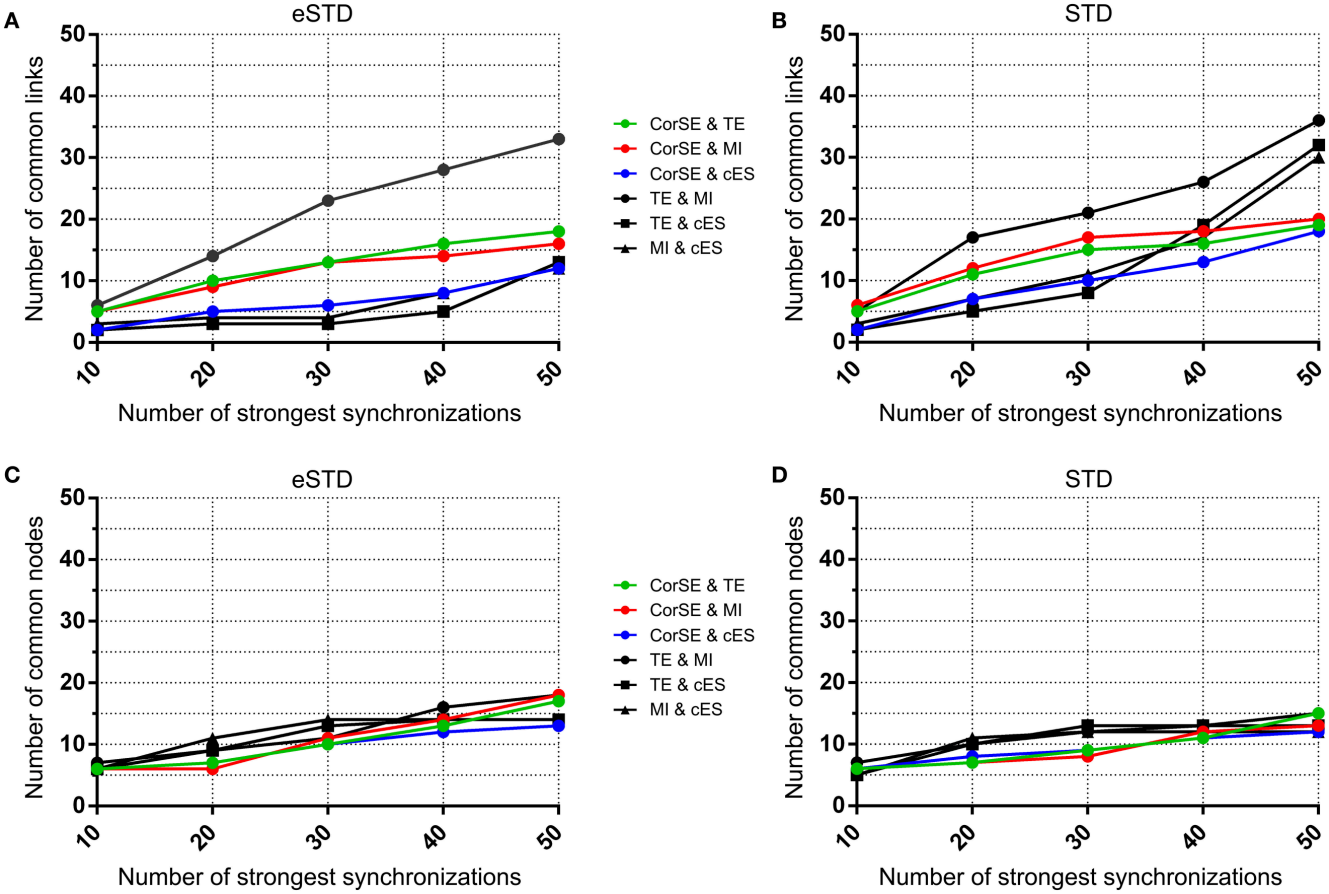
**The number of common nodes (channels) and the number of common links (pairwise synchronizations) as a function of the number of synchronizations for MEA2**. The number of common links found by the different algorithms using **(A)** eSTD and **(B)** STD based spike detection. The number of common nodes found by different algorithms using **(C)** eSTD and **(D)** STD based spike detection.

We present the results of different synchronization assessment algorithms applied on rat cortical network measurement data (MEA1 and MEA 2) in different forms: First, we demonstrate the obtained synchronization values for the different methods as adjacency matrices (Figures 5, 6). Then, the most synchronized channel pairs found with different methods are presented (Figures 7, 8) on the MEA layout. Finally, we compare the most synchronized channel pairs found by different algorithms (Figures 9, 10). To see the effects of the different spike detection methods, the results employing both spike detection methods (STD and eSTD) are presented for the event based algorithms (Figures 5–10).

Adjacency matrices present an overview of the found synchronicities. For MEA1, the highest synchronization was found between the channels 56 and 67 (represented in Figure 5 as (5,6) and (6,7), respectively, in the sequel similarly) by CorSE, and the TE (with eSTD) and MI (with eSTD) methods (Figures 5B–D). The rest of the methods resulted in different maximally synchronized culture locations. For MEA2, the highest synchronization was found between the channels 73 (7,3) and 83 (8,3) by MI (with STD and eSTD) and cES (with STD and eSTD) methods (Figures 6D,E).

For further comparisons between the algorithms and to demonstrate the most synchronized channel pairs (strongest synchronizations), the 40 strongest synchronizations for MEA1, and the 20 strongest synchronizations for MEA2, are shown in Figures 7, 8, respectively. The different algorithms found most synchronized channel pairs differently. Moreover, the different spike detection methods change the results of the event based synchronization assessment algorithms as can be seen also from Figures 5, 6. Among all algorithms considered, the results of the TE and MI methods are most similar where CorSE has more common outputs with both the TE and MI methods compared to the output of the cES method. For example, there are channels with more links than the others; in other words, network locations acting as hubs (see Figures 7, 8), such as channels 56 and 67 found by the TE and MI methods (with both spike detection methods) and CorSE as the top two channels with the highest numbers of links for MEA1. The cES method found channels 55 and 27 (with eSTD), and 83 and 51 (with STD) as the top two hub channels. For MEA2, channel 75 was a channel that could be considered a hub found by the TE and MI methods (with both spike detection methods) and CorSE, but again not by the cES method.

To assess the common aspects of the results produced with the difference algorithms in more detail, the number of common nodes (channels) and common links (pairwise synchronizations) are plotted according to the number of strongest synchronizations (Figures 9, 10). The results show that for MEA1 and using eSTD, all the algorithms found approximately a similar number of common nodes (Figure 9C), alike for MEA2 with either STD or eSTD (Figures 10C,D). However, the numbers of found common links between the common nodes show more dependence on the method used (Figures 10A,B). The results from the TE and MI methods had the most common links, and CorSE found more common links with these two algorithms than the cES algorithm did.

**Figure 11:**
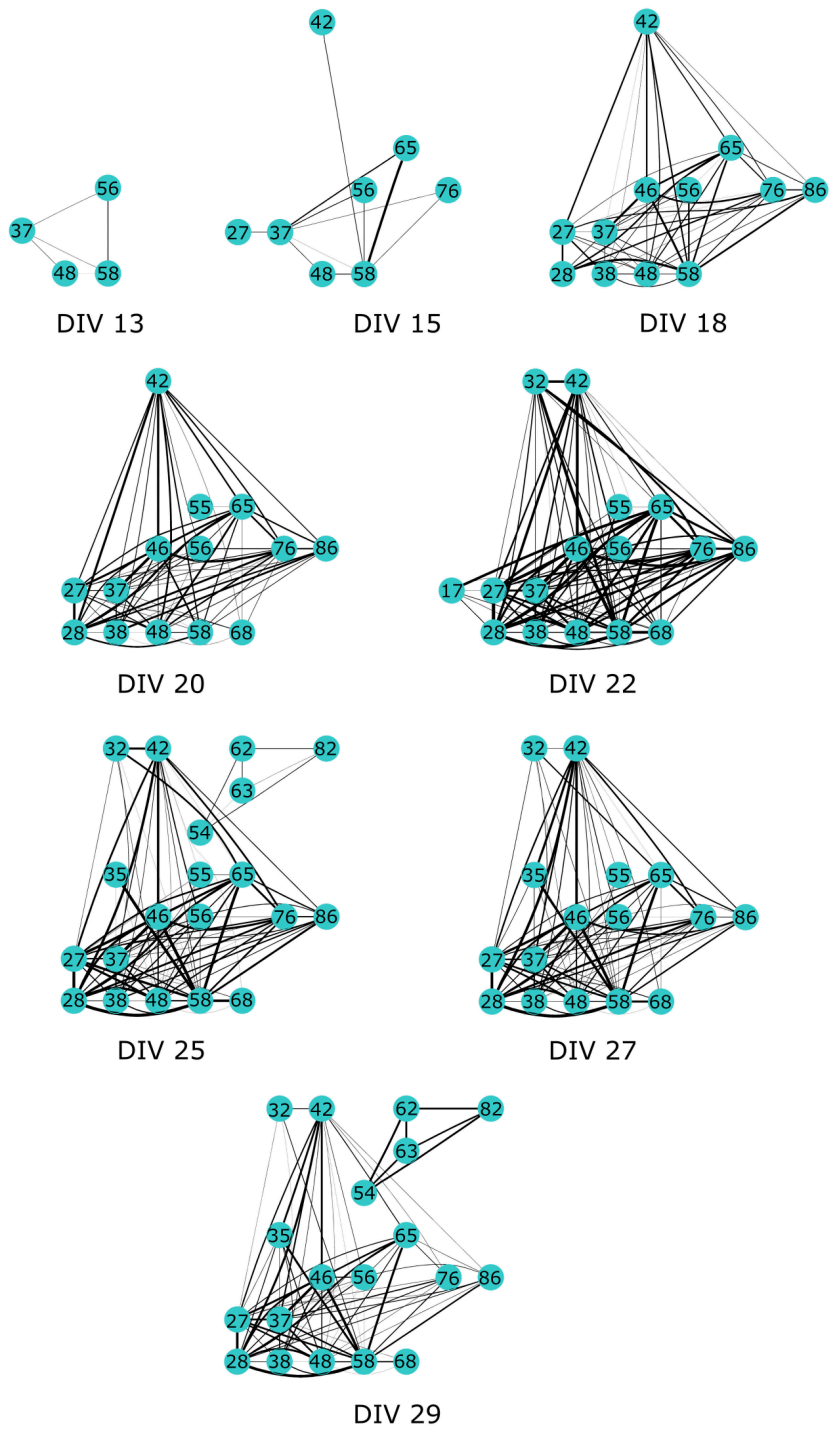
**The development of a functional neuronal network in a mouse cortical cell culture unraveled by CorSE**. The development of the network is shown between the 13th and 29th DIV showing the channels with pair-wise synchronizations *CorSE*_*xy*_ > 0.5.

**Figure 12:**
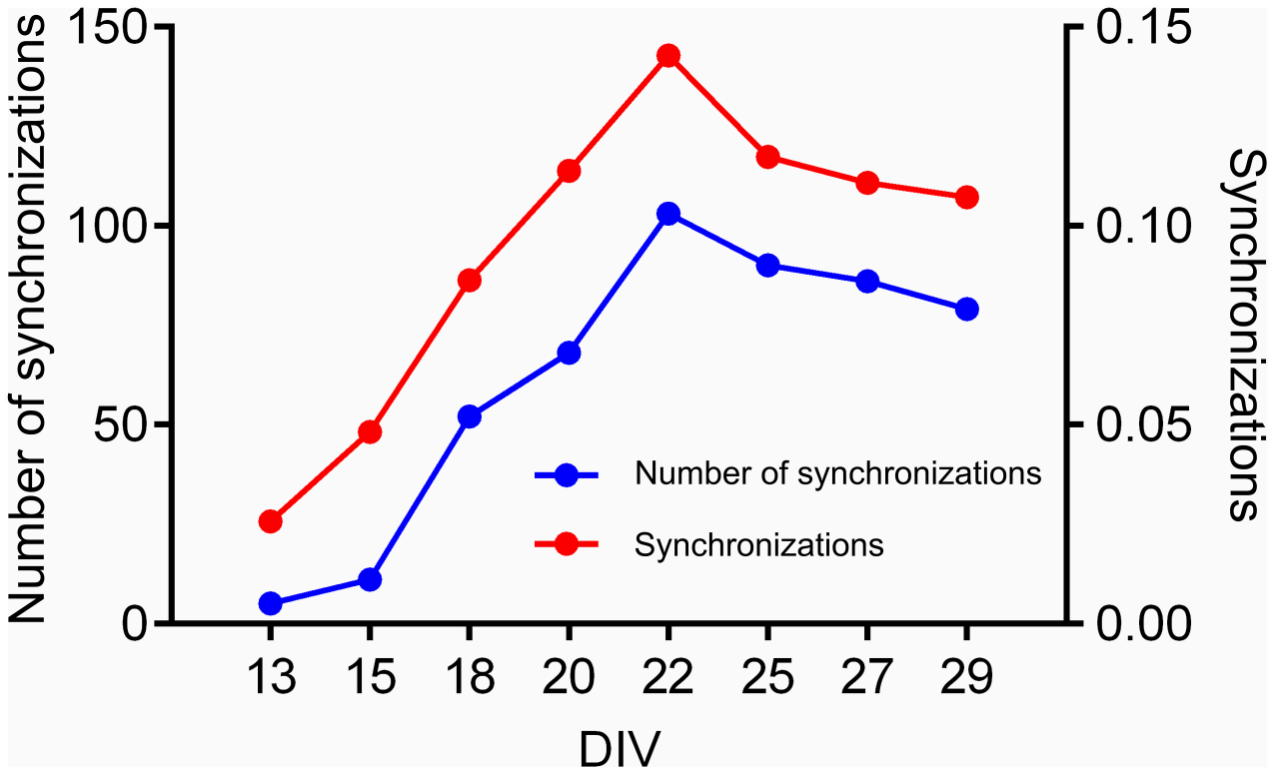
**The number of strong synchronizations and the mean overall synchronization found by CorSE in a developing mouse cortical cell network**. The number of synchronizations with *CorSE*_*xy*_ > 0.5 (blue) (left vertical axis), and the mean synchronizations between all channels (1770 links in total) (red) found at the difference measurement days (right vertical axis).

Finally, we present the results of CorSE in unraveling a developing mouse cortical neuron network. Figure 11 presents the development of a network between the 13th and 29th DIV. Channel pairs with synchronizations exceeding an arbitrary threshold (*CorSE* > 0.5, selected for illustrative purposes) are illustrated. The first links were seen on the 13th DIV, and thereafter the network gradually expanded while also synchronizations between all the channels were getting stronger, with the strongest network synchronizations found on the 22nd DIV (see Figure 12). The mean values of synchronizations between all the channel pairs (in total 1770 links) were calculated during the development of the network (see Figure 12). The results show that the mean values of all the synchronizations were also correlated with the number of channel pairs with synchronizations greater than *CorSE* > 0.5; in other words, the overall network synchronization strength followed the same trend with the observed channel pairs. Synchronization between some channels varied for different measurement days. A noteworthy example for this case is the smaller network that appeared on the 25th DIV, could not be observed on the 27th DIV, and reappeared on the 29th DIV with a stronger synchronization (Figure 11).

## Discussion

We have previously shown the feasibility of our correlated spectral entropy method CorSE for the assessment of synchronization (Kapucu et al., 2016a). In this paper, we have not only presented the usability of the method with larger real data, but we have made simulations justifying our concept and algorithm. For that, we realized both a toy simulation and simulations based on a widely used computation neuronal network model. The results of the simulations showed that it was possible to distinguish and assess the synchronization and connectivity strengths in neuronal populations by assessing the synchronization via the time correlated complexity measure CorSE.

In practice, in addition to biological sources, many other sources contribute to the measured electrophysiological signals. Any sources of noise that are not biological, e.g., artifacts and electrical interference, might influence the results of the algorithms employed. To assess this, we also performed the simulations with additive white Gaussian noise. Increasing the level of noise naturally had a negative effect on the results, as expected. Clearly, with the increasing noise level, more spikes were missed during spike detection, and the employed event based synchronization measures were more likely to fail. Concerning CorSE, increasing the level of noise had an effect on the uniformity of the spectral distribution, and thus on the synchronization measure. However, simulations showed that even with the increasing noise, it was still possible to distinguish between the connected and unconnected populations, even though the level of connectivity strength was not distinguishable anymore. Here, it may be noted that in all the event based synchronization assessment algorithms in the literature, anything other than detectable spikes is generally considered as biological “noise” (Obien et al., 2015), and a reasonable amount of information from LFPs is omitted. CorSE takes also LFPs into account in synchronization assessment. Thus, we observed the effects of different power ratios of EAPs and LFPs on the detected synchronization: the results showed that CorSE can assess the level of connectivity under the effects of synchronized LFPs. In contrary, since LFPs are considered as biological “noise” by the event based analysis, signals with different power ratios of EAPs and LFPs affect the spike detection accuracy, and thus the results of these algorithms.

For the rat cortical cells used in this study, the mutual information and transfer entropy algorithms showed the most corroborative results, as expected, since the two methods are based on the same theoretical grounds. CorSE exhibited common findings with the mutual information and transfer entropy algorithms, but the results from the corrected event synchronization method were generally different from the results by the rest of the algorithms. Since the corrected event synchronization method is spike rate adaptive by its nature, one possible explanation could be that its results were significantly affected by spike detection.

The similarities and differences of the considered algorithms can be summarized by joint evaluation of adjacency matrices (Figures 5, 6), by observing the differences in the most synchronized MEA channel pairs (Figures 7, 8), and in the common links and nodes (Figures 9, 10): CorSE, and the mutual information and transfer entropy algorithms all found the same link as the most synchronized link for MEA1, whereas for MEA2, the same most synchronized link was found by the mutual information and corrected event synchronization methods. Additionally, channels which had the highest number of links (hub-channels) were identified similarly by CorSE, the mutual information and transfer entropy methods. Consequently, it may well be that the most synchronized links could be found similarly by the different algorithms employed in this work, whereas the most differences might be found in the weaker synchronized links. In fact, the numbers of common links and nodes presented in Figures 9, 10 show similar results for the strongest 10 synchronizations, whereas the difference grows with the increasing number of strongest synchronizations. It is to be noted that since we assessed unguided neuronal cells which developed freely on the MEA plates, there is no ground truth about the network structures which the cell cultures formed. Thus, actual validation of the synchronies measured by different algorithms was impossible with the real data, but has been to an extent provided by the simulations. Still, the comparisons of the findings from the real data give an idea of the general usability of the algorithm, and of its biological plausibility.

Also the feasibility of CorSE to track neuronal development by means of synchronization is studied in this paper. We tracked developing network synchronization for several measurement days using an arbitrary synchronization detection threshold, here, *CorSE* > 0.5. This provided a clear view to the appearance of the functional network (Figure 11), although setting such a clear-cut threshold might have the effect of the temporary appearance and disappearance of some channels close to the detection threshold in the network development map (see Figure 11, 22–27 days *in vitro*). The results also correlated with the overall network synchronization behavior (Figure 12).

In conclusion, we have shown that CorSE is a promising tool to assess synchronization in neuronal networks. The method does not possess the shortcomings of event based methods resulting from possible poor event, e.g., spike, detection performance. Moreover, CorSE does not need specified effective frequency bands for the analysis. Our method would be useful especially for the acute analysis of possibly noisy recordings collected with MEAs for the experiments where fast processing is necessary: since it is based on the efficient fast Fourier transform and simple cross correlation, it can be used even for online processing (Semmlow and Griffel, 2014). In fact, for more than a decade, spectral entropy has been utilized in real-time electroencephalogram monitoring to quickly assess the of depth of anesthesia (Viertiö-Oja et al., 2004). We believe that methods not based on events, i.e., methods using all data recorded from the electrodes, such as CorSE, can help us in obtaining more information from the valuable neuronal network measurements, and provide robust synchrony measures, both *in vitro* and *in vivo*.

The Matlab code for CorSE has been developed to be applied straight forward on any time series data, and is publicly freely available in the Matlab Central File Exchange (https://se.mathworks.com/matlabcentral/fileexchange/59626-spectral-entropy-based-neuronal-network-synchronization-analysis—corse).

## Author contributions

FK developed the method, implemented the program code, designed and made the simulations, contributed to the culturing of mouse cortical cells, analyzed the data, and wrote the manuscript. IV made the computational neuronal network simulations, contributed to culturing of mouse cortical cells, and to the writing of the manuscript. JM cultured rat cortical cells, collected the data, and contributed to writing of the manuscript. CL contributed to method development and implementation. KL contributed to culturing of mouse cortical cells and writing of manuscript. JT contributed to method development and to the writing of the manuscript. JH contributed to method development and to the writing of the manuscript. All the authors approved the final version to be published.

## Funding

The work of FK has been supported by the 3DNeuroN project in the European Union's Seventh Framework Programme, Future and Emerging Technologies, grant agreement no. 296590; by the Academy of Finland under the project Bio-integrated Software Development for Adaptive Sensor Networks, project number 278882; by the Human Spare Parts Project funded by Tekes—The Finnish Funding Agency for Innovation; and by the Ella and Georg Ehrnrooth Foundation, Finland. The work of IV has been supported by the 3DNeuroN project in the European Union's Seventh Framework Programme, Future and Emerging Technologies, grant agreement no. 296590; by the Human Spare Parts Project funded by Tekes—The Finnish Funding Agency for Innovation; and by the Jane and Aatos Erkko Foundation, Finland, under the project Biological Neuronal Communications and Computing with ICT. The work of KL has been supported by the 3DNeuroN project in the European Union's Seventh Framework Programme, Future and Emerging Technologies, grant agreement no. 296590, and by the Human Spare Parts Project funded by Tekes—The Finnish Funding Agency for Innovation. The work of JT has been supported by the 3DNeuroN project in the European Union's Seventh Framework Programme, Future and Emerging Technologies, grant agreement no. 296590; and by the Jane and Aatos Erkko Foundation, Finland, under the project Biological Neuronal Communications and Computing with ICT.

### Conflict of interest statement

The authors declare that the research was conducted in the absence of any commercial or financial relationships that could be construed as a potential conflict of interest.

## Abbreviations

cES: corrected event synchronization
CorSE: correlated spectral entropy method
DIV: days *in vitro*
EAP: extracellular action potentials
EEG: electroencephalogram
ES: event synchronization
eSTD: thresholding at five times the standard deviation of the background noise
FN: false negative
LFP: local field potential
MEA: microelectrode array
MI: mutual information
SE: spectral entropy
STD: thresholding at five times the standard deviation of the signal
TE: transfer entropy
WGN: white Gaussian noise.
