# Corrigendum: Spectral Entropy Based Neuronal Network Synchronization Analysis Based on Microelectrode Array Measurements

**DOI:** 10.3389/fncom.2020.586506

**Published:** 2020-11-04

**Authors:** Fikret E. Kapucu, Inkeri Välkki, Jarno E. Mikkonen, Chiara Leone, Kerstin Lenk, Jarno M. A. Tanskanen, Jari A. K. Hyttinen

**Affiliations:** ^1^Department of Pervasive Computing, Tampere University of Technology, Tampere, Finland; ^2^Computational Biophysics and Imaging Group, Department of Electronics and Communication Engineering, BioMediTech, Tampere University of Technology, Tampere, Finland; ^3^Department of Psychology, Center for Interdisciplinary Brain Research, University of Jyväskylä, Jyväskylä, Finland; ^4^Department of Management and Production Engineering, Politecnico di Torino, Torino, Italy

**Keywords:** synchronization, spectral entropy, correlation, mouse cortical cells, rat cortical cells, developing neuronal networks, MEA, microelectrode array

In the original article, there was a mistake in the legend for **Figure 8** as published. The “MEA1” in the text was supposed to be “MEA2” in the legend. The corrected legend appears below.

Also, there was a mistake in Figures 7 and 8 as published. The figures were not representing the text in the original publication. Since the text is written according to the published data (which can be easily confirmed by the original Figures 5 and 6), the data is not well-represented in the Figures 7 and 8. The thickness of lines between the nodes were plotted wrong. Otherwise all the channels and connections between those channels are correct, except for Figure 7E cES (eSTD) and Figure 8E cES(STD). The original Figure 7E cES (eSTD) and Figure 8E cES(STD) were possibly plotted from the wrong data. The corrected Figures are now representing the originally published data and the text without any mistakes. The corrected Figures 7 and 8 appear below. The authors apologize for this error and state that this does not change the scientific conclusions of the article in any way, in fact the corrected Figures now firmly represent the results and conclusions presented in the original article.

**Figure 7:**
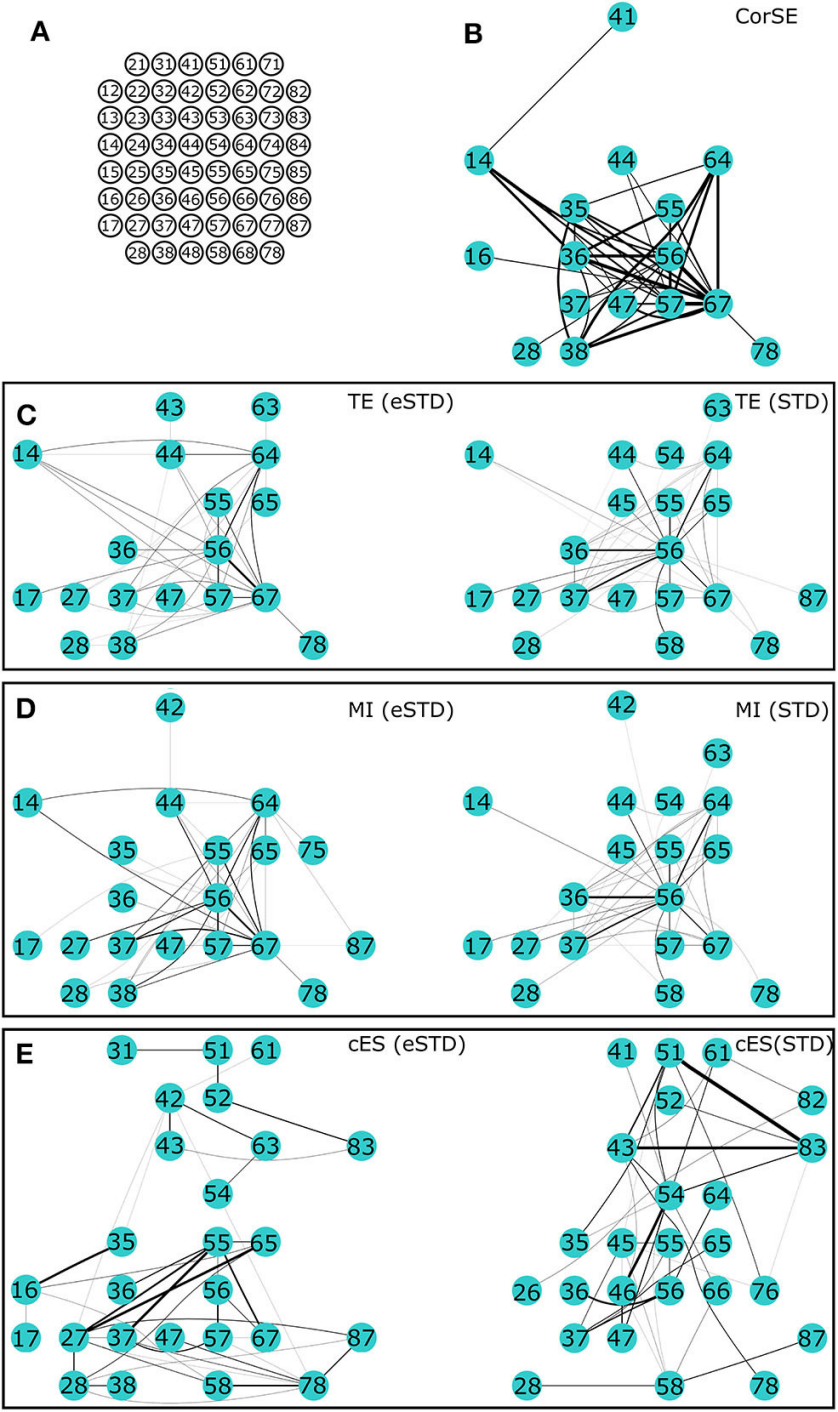
**The 40 most synchronized channel pairs (strongest synchronizations) calculated for MEA1. (A)** MEA layout. The strongest paired channels and their links found by **(B)** CorSE, **(C)** TE with eSTD (left) and with STD (right), **(D)** MI with eSTD (left) and with STD (right), and **(E)** cES with eSTD (left) and with STD (right).

**Figure 8:**
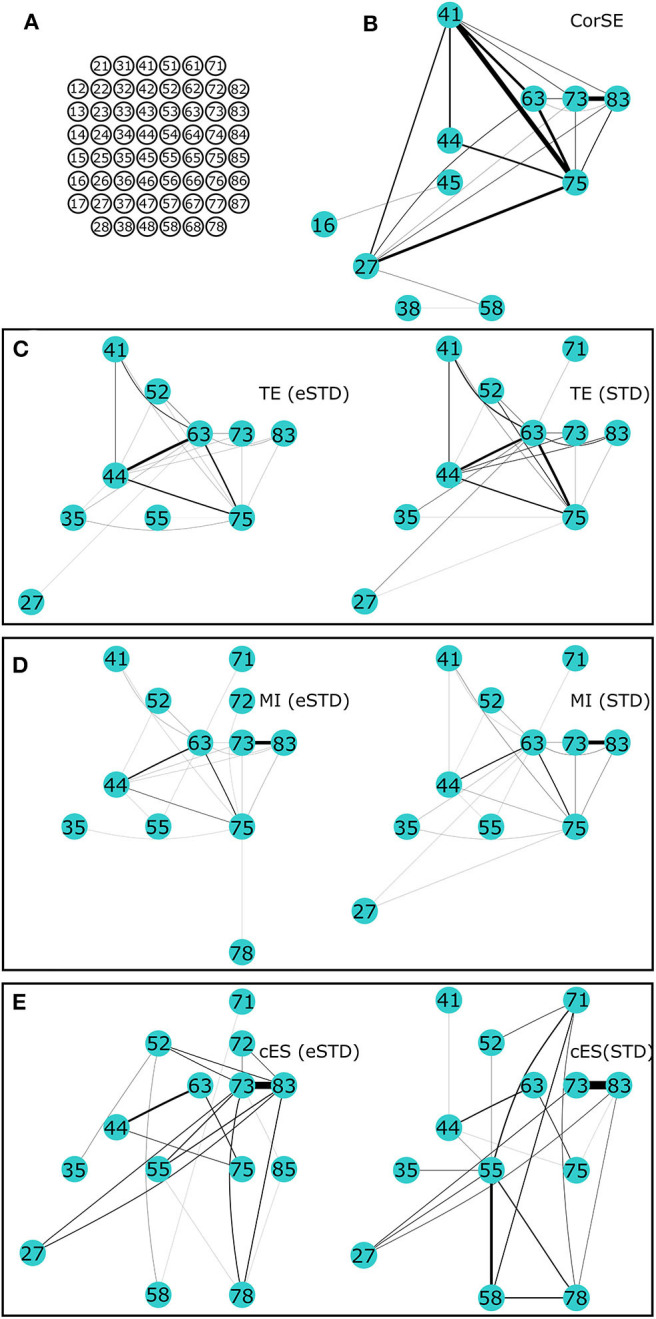
**The 20 most synchronized channel pairs (strongest synchronizations) calculated for MEA2. (A)** MEA layout. The strongest paired channels and their links found by **(B)** CorSE, **(C)** TE with eSTD (left) and with STD (right), **(D)** MI with eSTD (left) and with STD (right), and **(E)** cES with eSTD (left) and with STD (right).

Additionally, in the original article, there was an error. In the following sentence, the “CorSE” method should not be included:

“For MEA2, the highest synchronization was found between the channels 73 (7,3) and 83 (8,3) by CorSE, and the MI (with STD and eSTD) and cES (with STD and eSTD) methods (Figures 6B,D,E).”

A correction has been made to Results section, Sub-section MEA recordings, the second paragraph:

For MEA2, the highest synchronization was found between the channels 73 (7,3) and 83 (8,3) by MI (with STD and eSTD) and cES (with STD and eSTD) methods (Figures 6D,E).

Relatedly, the same error is seen also in Discussion. In the following sentence, the “CorSE” method for MEA2 should not be included:

“CorSE, and the mutual information and transfer entropy algorithms all found the same link as the most synchronized link for MEA1, whereas for MEA2, the same most synchronized link was found by CorSE, and the mutual information and corrected event synchronization methods.”

A correction has been made to the Discussion section, the fourth paragraph:

CorSE, and the mutual information and transfer entropy algorithms all found the same link as the most synchronized link for MEA1, whereas for MEA2, the same most synchronized link was found by the mutual information and corrected event synchronization methods.

The authors apologize for these errors and state that they do not change the scientific conclusions of the article in any way. The original article has been updated.

